# Functional Neural Networks in Writer's Cramp as Determined by Graph-Theoretical Analysis

**DOI:** 10.3389/fneur.2021.744503

**Published:** 2021-11-23

**Authors:** Jana Schill, Kirsten E. Zeuner, Arne Knutzen, Inken Tödt, Kristina Simonyan, Karsten Witt

**Affiliations:** ^1^Department of Neurology, School of Medicine and Health Sciences, University of Oldenburg, Oldenburg, Germany; ^2^Department of Otolaryngology – Head & Neck Surgery, Harvard Medical School, Boston, MA, United States; ^3^Department of Otolaryngology – Head & Neck Surgery, Massachusetts Eye and Ear, Boston, MA, United States; ^4^Department of Neurology, Christian Albrechts University, Kiel, Germany; ^5^Research Center Neurosensory Science, University of Oldenburg, Oldenburg, Germany

**Keywords:** writer's cramp, functional magnetic resonance imaging (fMRI), functional brain connectivity, cerebellum, network analysis, dystonia

## Abstract

Dystonia, a debilitating neurological movement disorder, is characterized by involuntary muscle contractions and develops from a complex pathophysiology. Graph theoretical analysis approaches have been employed to investigate functional network changes in patients with different forms of dystonia. In this study, we aimed to characterize the abnormal brain connectivity underlying writer's cramp, a focal hand dystonia. To this end, we examined functional magnetic resonance scans of 20 writer's cramp patients (11 females/nine males) and 26 healthy controls (10 females/16 males) performing a sequential finger tapping task with their non-dominant (and for patients non-dystonic) hand. Functional connectivity matrices were used to determine group averaged brain networks. Our data suggest that in their neuronal network writer's cramp patients recruited fewer regions that were functionally more segregated. However, this did not impair the network's efficiency for information transfer. A hub analysis revealed alterations in communication patterns of the primary motor cortex, the thalamus and the cerebellum. As we did not observe any differences in motor outcome between groups, we assume that these network changes constitute compensatory rerouting within the patient network. In a secondary analysis, we compared patients with simple writer's cramp (only affecting the hand while writing) and those with complex writer's cramp (affecting the hand also during other fine motor tasks). We found abnormal cerebellar connectivity in the simple writer's cramp group, which was less prominent in complex writer's cramp. Our preliminary findings suggest that longitudinal research concerning cerebellar connectivity during WC progression could provide insight on early compensatory mechanisms in WC.

## Introduction

Dystonia is the third most common type of movement disorder (1). It includes heterogeneous symptoms characterized by involuntary muscle contractions resulting in abnormal movement patterns (2). In the case of writer's cramp (WC), dystonic symptoms affect the dominant hand during writing or, in a more complex form of the disease, during additional fine motor tasks (3). While the different forms of dystonia have been clinically well-characterized, the cause of WC remains unclear.

Several studies demonstrated a loss of surround inhibition in the primary motor cortex and abnormal basal ganglia activity (4–7). Furthermore, a sensory disorder has been postulated, including maladaptive cortical plasticity with dedifferentiation of somatosensory representations (8, 9), abnormal temporal discrimination (10), impaired cortical somatosensory processing and abnormal sensorimotor integration (11, 12).

In the past years it has become increasingly evident that many brain functions rely on complex interactions between different brain regions. Mathematical approaches, including those of theoretical graph analysis, have been employed to study brain networks (13). Many brain disorders have been attributed to changes in network function, for example Alzheimer's dementia, Parkinson's disease and Huntington's disease (14–16).

Fuertinger and Simonyan (17) showed that the neuronal network in spasmodic dysphonia not only differs from that of healthy volunteers, but also shows differences in network organization between clinical subtypes. Network analysis has been employed to investigate differences between task-specific and non-task-specific dystonias, suggesting the presence of large-scale network changes as a common feature in isolated focal dystonias (18). It has been shown that a feature shared between task-specific dystonias was disorganization of their network kernels, i.e., those nodes which play the most important communicative role in the network (19). These studies highlight the aspect that task specific dystonias are network disorders. Taken together, these studies also illustrate that different methods of network analyses can provide synergistic knowledge about the pathophysiological changes in focal dystonias.

Our main objective was to determine whether changes in the motor network in WC are persistent, that is, whether they are present even during periods in which no motor symptoms occur. Therefore, we employed a finger tapping task with the unaffected left hand in WC patients and included only those who were not experiencing symptoms during task performance. As writer's cramp is a movement disorder affecting skilled hand motor control, we chose finger tapping as a robust paradigm to elicit activity in the neural network of hand motor control. By only involving the non-symptomatic hand, we ensured that the resulting brain activation reflected underlying, persistent disease-inherent changes, in contrast to changes brought about by symptomatic differences in motor outcome. Our working group has already published two analyses on this data set: In a first analysis, we were able to show that WC patients have abnormal grey matter volume in the putamen and globus pallidus, both bilaterally. Furthermore, functional analysis revealed reduced activity in the right putamen and left globus pallidus (20). Interestingly, only basal ganglia abnormalities, but no cerebellar changes were found. Extending these findings, in a second analysis we employed a dynamic causal modelling (DCM) approach to investigate abnormal effective connectivity (11). In addition to a dysfunctional cortico-basal ganglia motor network, we also found abnormal connectivity in the cerebello-cortical motor network. In the current study, we used the same data set in a graph theoretical network analysis, to investigate firstly, whether our previous findings can be replicated with another approach and, secondly, to establish whether there are whole-brain functional connectivity changes underlying WC. While our DCM analysis was restricted to predefined network, this graph-theoretical approach allows for a holistic, data-driven assessment of the brain connectivity.

We generated the whole-brain network from functional MRI (fMRI) data to compare the contributions of different brain areas to the network function in patients vs. healthy controls. In a secondary, exploratory analysis (due to small sample size), we divided our patient group in those with simple writer's cramp (SWC), that only affected the hand during writing, and those with complex writer's cramp (CWC), which also affected other fine motor tasks. We then compared their respective networks using the same methods employed for our primary analysis.

We hypothesized that the functional whole-brain network of writer's cramp patients would be altered even in the absence of motor symptoms. We based this hypothesis on findings of resting state dystonia research, which report network alterations when no task was performed during scanning (18, 19), and on our own research finding network alterations during a non-symptomatic motor task (11). We also hypothesize that communication between network modules (i.e., communities of regions working together) is impaired in WC, because previous research reporting impairment of motor inhibition and abnormal sensorimotor integration leads us to believe that connectivity between regions is altered in WC. Lastly, as our DCM study revealed dysfunction of the cortico-basal ganglia motor network and the cerebello-cortical motor network, we specifically expected abnormal connectivity of the basal ganglia and the cerebellum.

## Methods

### Subjects

A total of 20 WC patients (46.5 ± 13.3 years of age, 11 females/nine males) and 26 healthy controls (49.6 ± 8.8 years of age, 10 females/16 males) participated in the study. Sixteen patients and 24 healthy controls of this sample had already been analyzed in our previous studies (11, 20). Nine patients presented with simple writer's cramp (SWC) of their right hand, which was symptomatic only during writing, while 11 patients were diagnosed with complex writer's cramp (CWC), which affected their right hand also during other fine motor tasks, including buttoning, using the computer mouse and picking up small objects. Diagnosis and subtype classification were established by a neurologist. To this end, the Writer's Cramp Rating Scale (21) and the Arm Dystonia Disability Scale (22) as well as the patient's medical history were employed. All participants were right-handed according to the Edinburgh Handedness Inventory (23). Exclusion criteria included any neurological or psychiatric disorders other than writer's cramp. Musicians and professional typists were excluded from the study. The last botulinum toxin injection was performed at least three months before study inclusion. Clinical characteristics of patients are shown in Table 1.

**Table 1:** Participant demographics.

	**Controls**	**Patients**			***p***	
		**All**	**SWC**	**CWC**	**Patients vs. Controls**	**SWC vs. CWC**
Number of participants	26	20	9	11	-	-
Gender (female/male)	10/16	11/9	5/4	6/5	0.264	0.964
Age (y, mean ±SD)	49.6 ± 8.8	46.5 ± 13.3	45.6 ± 15.2	47.2 ± 12.3	0.371	0.794
Handedness	Right	Right	Right	Right	-	-
Symptom duration (y, mean ± SD)	n.a.	13.2 ± 8.9	9.1 ± 6.6	16.6 ± 9.4	-	0.060
BoNT treatment (yes/no)*	n.a.	11/9	4/5	7/4	-	
Last BoNT injection (months, mean ± SD)**	n.a.	36.5 ± 34.3	20.8 ± 26.6	45.4 ± 39.3	-	0.297
WCRS score (mean ± SD)	n.a.	10.9 ±5.5	10.2 ± 5.2	11.4 ± 5.8	-	0.654
ADDS score (mean ± SD)	n.a.	61.1 ± 12.9	66.7 ± 10.3	56.6 ± 13.5	-	0.083

*P-values were computed via independent t-tests, except for gender (chi-squared test). ADDS, Arm Dystonia Disability Scale; WCRS, Writer's Cramp Rating Scale; n.a., not applicable.*

*Last injection was at least 3 months before study date.

**Only for those patients that had received BoNT treatment.

### Experimental Design

Participants performed a finger tapping task while lying in the MRI scanner. The task consisted of pressing four keys in a specific sequence. All participants used their left hand. This was the non-dominant hand and, for all patients, it was non-dystonic. Fingers were numbered from 2 to 5, excluding the thumb and starting with the index finger (position 2) up to the little finger (position 5). Participants were instructed to perform the sequence 5–2–4–3–5 repeatedly as quickly and accurately as possible. Prior to the scan, task understanding was tested by a few trial sequences. During the scan, the sequence was displayed visually via a mirror to minimize working memory load. A trial block consisted of 30 s tapping followed by 30 s rest. A total of 15 blocks had to be performed. Participants were fixating on a cross, which turned into a circle for 100 ms after each key press. Key presses were recorded for behavioral analysis. All patients were observed during task performance to ensure that no dystonic symptoms, including mirror movements, occurred.

### Behavioral Analysis

Task performance was assessed in terms of correct tapping sequences and tapping speed (number of taps per block). Two repeated-measures ANOVA were used, each with one within-subjects factor (number of blocks) and one between-subjects factor (group). Statistical significance was determined by a threshold of *p* < 0.05 corrected for multiple comparisons by a Bonferroni correction, yielding *p* < 0.025.

### Image Acquisition and Processing

Images were acquired on a 3T whole-body MRI scanner (Achieva; Philips, Best, the Netherlands) at the Neurocenter of the Christian Albrechts University Hospital. An 8-channel head coil was used. Stimulus presentation and response recording were achieved via an IFIS system (*Invivo*, Gainesville, FL). Functional scans were obtained using a whole-brain echo planar imaging (EPI) sequence consisting of 360 volumes of 38 slices each. Axial images were aligned to the anterior-posterior plane. The following parameters were used: *TR* = 2500 ms, *TE* = 36.4 ms, slice thickness = 3 mm, intersclice gap = 0.3 mm, FOV = 216 x 216 x 125.1 mm^3^, matrix = 64 x 64, flip angle = 90 degree, voxel size = 3.375 x 3.375 x 3 mm^3^. Furthermore, each participant received a 3D T1-weighted gradient echo structural MRI scan with sagittal volume excitation. The following parameters were used: *TR* = 7.8 ms, *TE* = 3.6 ms, *TI* = 800 ms, FOV = 160 x 240 x 240 mm^3^, matrix = 256 x 256, flip angle = 8 degree, slices = 160, voxel size = 1 x 0.94 x 0.94 mm^3^.

Data preprocessing was performed using a standard AFNI imaging preprocessing pipeline. For this, afni_proc.py was used to generate preprocessing scripts that were consistent between all subjects. The scripts included the following blocks: despike, tshift, align, volreg, blur, mask, and scale. Thereby, EPI scans were despiked using AFNI's 3dDespike with standard settings. They were slice time corrected using a Fourier interpolation. Then, scans were registered to the first volume and normalized to Taillarach space. After spatial smoothing using a 4-mm Gaussian kernel, the voxel time series were scaled such that their mean was set to 100 (this is achieved by the afni_proc.py -scale block and allows for better comparability of time series between scans). Volumes containing differential movement >0.5 mm per TR were censored during subsequent analysis steps, meaning they were removed from the data set. No interpolation was applied. The scaled files were fed into the network analysis.

### Functional Network Construction

The network construction pipeline employed here was adapted from Fuertinger et al. (24) and Fuertinger and Simonyan (17). Codes were written in Python using the open-source libraries NumPy (25), SciPy (26) and Matplotlib (27).

The cytoarchitectonic maximum probability maps and macrolabel atlas (24, 28) was used to parcellate the whole brain into 212 regions of interest (ROIs). Thereby, 142 cortical, 36 subcortical and 34 cerebellar regions were included in the analysis. Voxelwise-averaged time series of the task-production fMRI were computed for every ROI in all participants. Only volumes acquired during the tapping blocks were used during subsequent steps. Pairwise regional interactions were calculated as normalized mutual information (NMI) coefficients. As described previously (17), NMI coefficients preserve the non-zero structure of the widely used pairwise Pearson correlation matrix but come with the advantage of non-negativity of all interactions. The NMI coefficients were obtained by dividing the classical mutual information (29) of two regions by the geometric mean of the associated Shannon entropies (30). This provides a statistical dependence measure scaled to the interval [0, 1], with 0 indicating statistical independence and 1 indication mutual dependence.

For each subject, a 212 x 212 whole-brain NMI matrix was computed and subsequently used to construct a weighted undirected graph, where ROIs acted as nodes and the NMI values provided the edge weights. Thereby, each graph represented the whole brain of a participant. For each graph, network density was calculated as the number of present edges (non-zero NMI) divided by the number of possible edges in the graph. All networks had a 100% density and were therefore thresholded. As very dense networks (density > 50%) tend towards random graph characteristics (31, 32), all networks were thresholded to 50% by iteratively removing (i.e.; setting to zero) the weakest edges of the graph until 50% density was reached. As a last step, nodes with fewer than 5% of all possible connections were also removed, as these were only sparsely connected and likely to be noise artifacts. Nodes that became thereby disconnected (i.e., nodes with all edges removed) were excluded from further analysis. Reduced individual graphs were computed for each group, which contained only nodes that were present in all subjects of that group. Then, a group-averaged network was computed for each group, yielding four networks (control and WC in the main analysis; and SWC and CWC in the secondary analysis). As these reduced networks again had a density of 100%, they were thresholded again to 50% (no nodes were disconnected in this last step). This thresholding approach (i.e.; the nodal elimination) resulted in networks with different network spaces for each group. We argue that these differences reflect disease-inherent changes in network function and therefore warrant interpretation. The network metrics investigated in this study are not affected by the nodal elimination approach, as they do not rely on the number of network nodes. They are, however, influenced by the network's density, which was adjusted to the same 50% in all groups.

### Graph Theoretical and Statistical Analysis

Network processing and visualization was programmed in Python, while the computation of optimal modular decompositions and network metrics was performed using MATLAB (The MathWorks, Inc.) and the Brain Connectivity Toolbox (33). BrainNet Viewer (34) was employed to generate 3D network images embedded in reference brain models.

#### Network Metrics

Four network metrics were computed: nodal degree, nodal strength, clustering coefficient and global efficiency. Nodal degree was calculated as the number of edges connected to a node. Nodal strength refers to the sum of the weights of all edges connected to a node. To ensure comparability between networks of different network spaces, both nodal degree and strength were normalized by dividing them by the number of nodes in the network. The clustering coefficient served as a measure of nodal segregation, as it illustrates the presence of functional cliques in a node's local neighborhood. The clustering coefficient is defined as the geometric mean of weights in triangles around the node. Network integration, on the other hand, was measured by means of global network efficiency, which is calculated as the average inverse shortest path length in the network. Inverse edge weights were used as connections lengths. A two-tailed non-parametric permutation *t*-test with 20,000 Monte Carlo randomizations was used to assess statistical significance of differences in these metrics between two groups (*p* < 0.05 corrected for multiple comparisons by a Bonferroni correction, yielding *p* < 0.0025). Permutation tests were the method of choice as they do not require assumptions concerning the distribution of the measure under investigation. Their only assumption is that the observations are independent, which was the case in this study.

#### Optimal Modular Decomposition

A graph community analysis was carried out to assess the global reconfiguration of the functional networks in different groups. An optimal modular decomposition divides a graph into non-overlapping groups of connected nodes, such that the number of within-module edges is maximized and the number of between-module edges is minimized. For each graph, the optimal modular decomposition was estimated by maximizing the Newman modularity (35). For this, a heuristic optimization strategy was employed that utilized the Kernighan-Lin algorithm (36). In the first step, each node was assigned its own module. This decomposition was then refined by employing the modularity maximization algorithm one-hundred times, which also accounted for the randomness of the approach (24). Based on the average nodal module assignment, the final modular decomposition was computed. Node 1 always served as reference to account for differences in module numbering due to randomness. Similarity of decompositions between groups was estimated using their partition distances (pd), which illustrate the normalized variation of information between two community affiliation vectors (37). Again, a non-parametric permutation *t* test was used to assess group differences. In a next step, the networks' spatial community structure and hub formation were compared.

#### Hub Formation

Any node with both degree and strength at least one standard deviation above the network's mean respective metric was considered a hub. Nodes that did not fulfill this criterion but were among the top 30% for both their strength and degree were considered as high-influence nodes. To distinguish between connector hubs (nodes connecting different communities) and provincial hubs (nodes connecting nodes within one community), the nodal participation coefficient *pc*_*i*_ was calculated (17, 38). The *pc*_*i*_ resembles the distribution of inter- vs. intra-module connections and is maximized when a node's edges are evenly distributed between the networks modules. Hubs with at least 90% of the theoretical maximum *pc*_*i*_ value of a network were considered as connector hubs, all remaining hubs were classified as provincial hubs. Hubs were assessed qualitatively for their number and spatial distribution within each network. Results will be reported in a descriptive manner, as the hub analysis does not allow for statistical testing.

## Results

### Task Performance

There were no significant differences between groups in the total number of taps per block [*F*_(1, 30)_ = 0.774, *p* = 0.38] and the number of correctly tapped sequences [*F*_(1, 30)_ = 1.045, *p* = 0.31]. Both groups improved over time in both measures [total taps: *F*_(4, 434)_ = 36.861, *p* < 0.001; correct taps: *F*_(6, 434)_ = 16.165, *p* < 0.001, Supplementary Figure 1]. Patients were asked whether they experienced dystonic symptoms during scanning, which they denied.

### Neural Network Analysis

The thresholding approach revealed that in the control network, 156 of 212 regions contributed to the network (i.e., were connected to at least one other node). For WC patients, this was true for 131 nodes. Within the overall WC group, the SWC network had 166 nodes, compared to 154 nodes in the CWC network.

#### Controls vs. Patients

##### Network Metrics

The permutation test showed a significant difference in mean clustering coefficient (Controls: 0.12 ± 0.02, WC Patients: 0.14 ± 0.02, *p* ≤ 0.00005) but not in mean nodal degree (Controls: 0.50 ± 0.17, Patients: 0.50 ± 0.18, *p* = 0.88), mean nodal strength (Controls: 0.08 ± 0.03, Patients: 0.09 ± 0.04, *p* = 0.07) or global efficiency (Controls: 0.15 ± 0.03, Patients: 0.16 ± 0.08, *p* = 0.46).

##### Hub Formation

**Figure 1:**
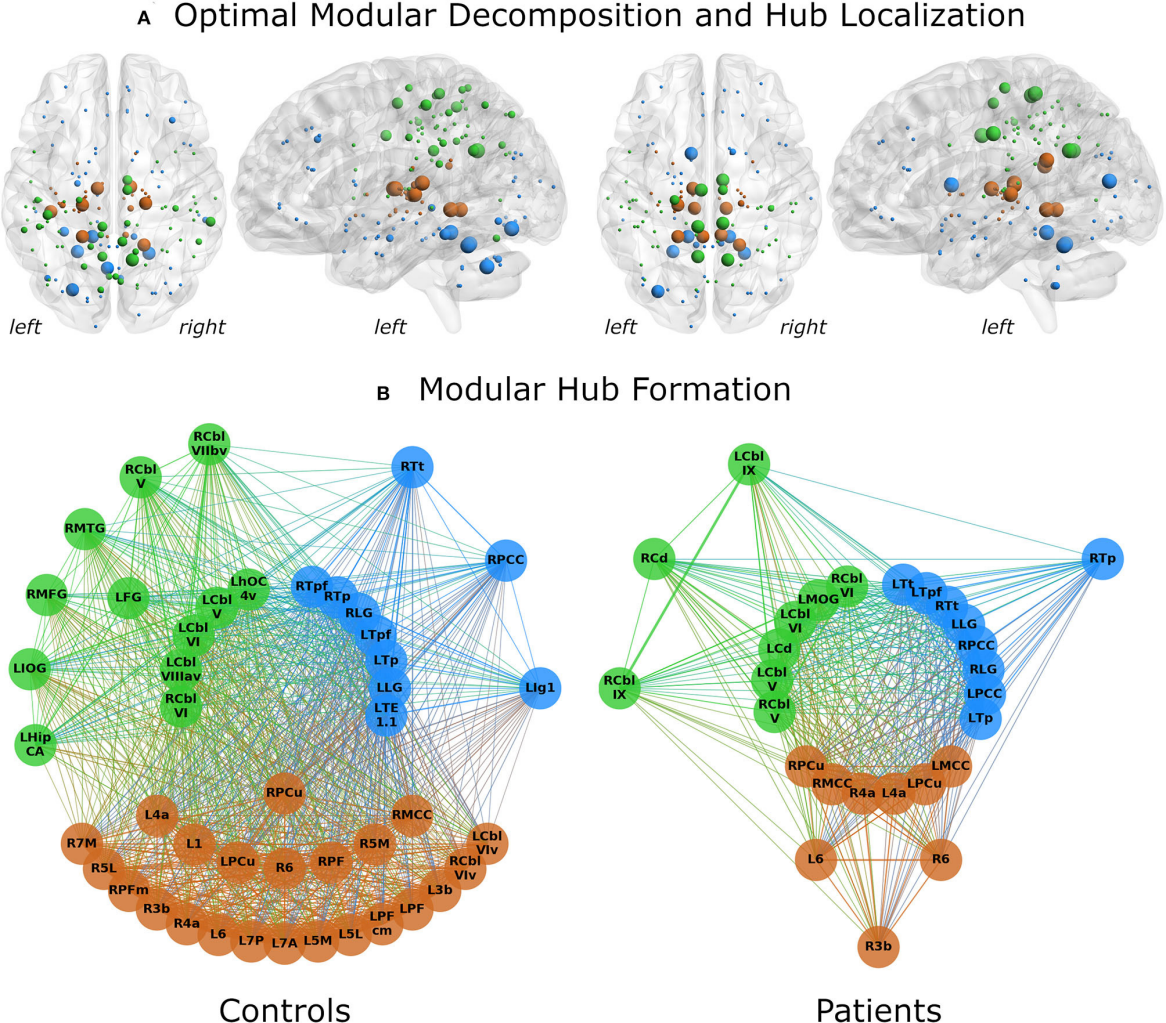
Network analysis results for control subjects (left) and patients (right). **(A)** The optimal modular decomposition is shown by colored nodes in a 3D glass brain. Both groups had three modules: a fronto-occipital module (blue), a parietal module (green) and a subcortical module (brown). The size of each node indicates its hub status, from largest to smallest: connector hubs, provincial hubs, high influence nodes, other nodes. In both groups, hub formation was mostly restricted to the posterior part of the brain. **(B)** Hubs per module. All regions that were either hubs or high influence nodes are presented as colored nodes. Color illustrated module affiliation (as in **A**). Nodes are arranged in three circles according to their hub status: connector hubs (innermost circle), provincial hubs (intermediate circle) and high influence nodes (outer circle). Controls recruited nearly the same number of hubs as patients, but these hubs communicated with more high influence nodes. 1/3b/6, BAs 1/3b/6; 4a, anterior part of BA 4; 5L/5M, subdivisions of BA 5; 7A/7M/7P, subdivisions of BA 7; Cbl V/VI/VIv/VIIbv/VIIIav/IX, cerebellar lobules V/VI/VI vermis/VIIb vermis/VIIIa vermis/IX; Cd, caudate nucleus; FG, fusiform gyrus; HipCA, subdivision of the hippocampus; hOC4v, ventral part of area hOC4; IOG/MOG, inferior/middle occipital gyrus; lg1, insular subdivision lg1; LG, lingual gyrus; MCC/PCC, middle/posterior cingulate cortex; MFG, middle frontal gyrus; MTG, middle temporal gyrus; PCu, precuneus; PF/PFcm/PFm, areas PF/PFcm/PFm in the inferior parietal cortex; TE1.1, area TE1.1; Tp/Tpf/Tt, parietal/prefrontal/temporal subdivision of the thalamus; L, left; R, right.

Both control and WC patient networks had an optimal modular decomposition comprised of three modules: One fronto-occipital community, which also included cerebellar nodes, one parietal community and one subcortical community (Figure 1A). As the control network had a higher total number of nodes (*N* = 156), its fronto-occipital and parietal modules included more nodes than those of the patient network. Modularization as measured by the partition distance pd was significantly different between groups (*pd* = 0.312, *p* < 0.0001).

Differences in hub formation included different utilization of hubs (connector vs. provincial), loss of hubs (degradation to high influence nodes or even absence of the node), or formation of new hubs.

While the number of hubs was similar in the control and the patient network (21 vs. 22), the control network contained fewer connector hubs (13 vs. 20). Notably, patients had only two provincial hubs, while controls had eight (Figure 1B). Patients showed a five-fold decrease in the number of high-influence nodes found in the control network (24 in controls vs. 5 in patients). Abnormal hub formation in WC patients included the cingulate gyrus, thalamus, and cerebellum.

**Figure 2:**
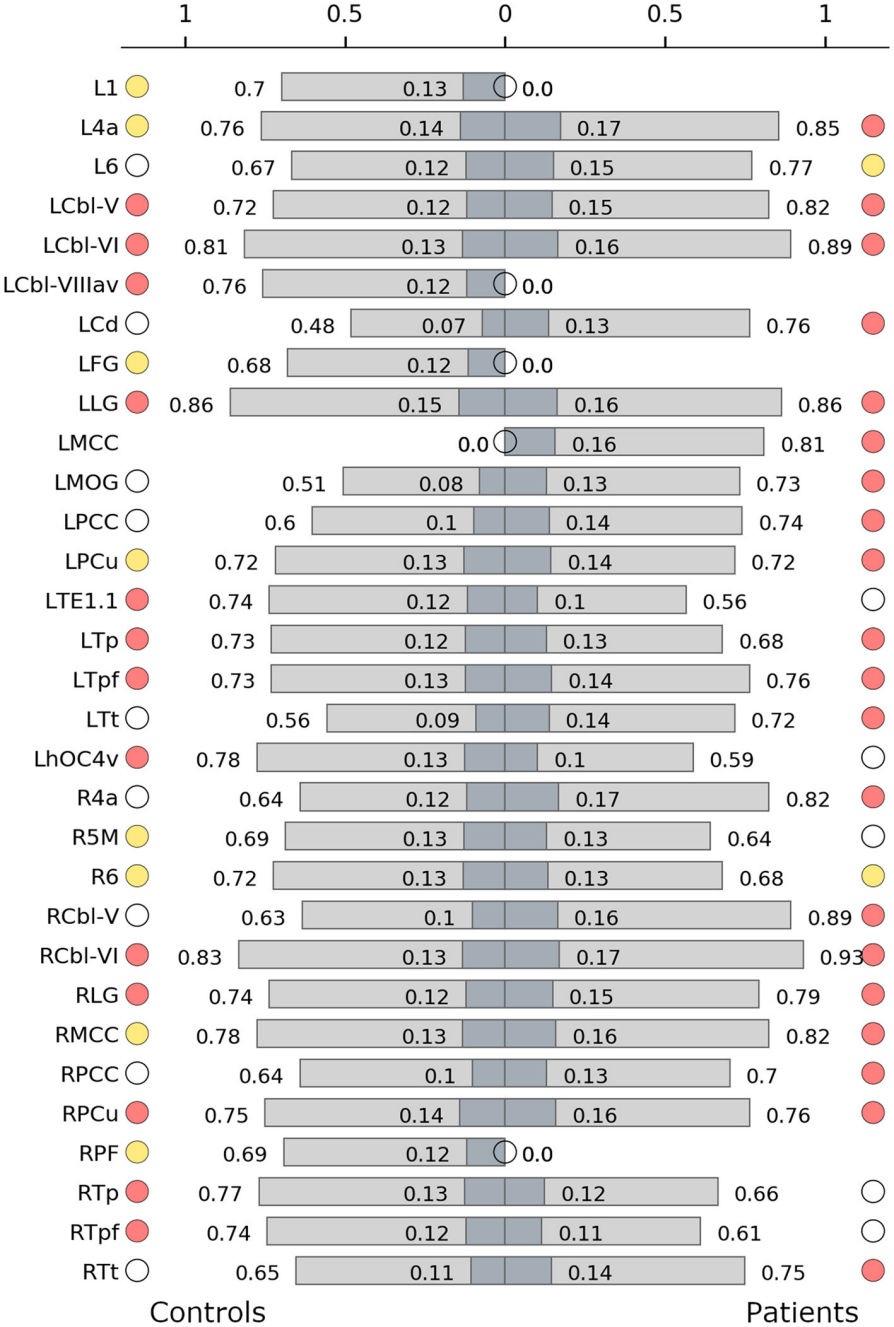
Hub characteristics of the control **(left)** and the patient network **(right)**. The graph shows all nodes that were recruited as hubs in either of the two networks. For each hub, normalized nodal degree (light grey) and normalized nodal strength (dark grey) are indicated by bars. White circles denote nodes that were not hubs in the respective network, absence of circles denotes that the node was not present in the respective network. Red circles denote connector hubs, yellow circles denote provincial hubs. 1/6, BAs 1/6; 4a, anterior part of BA 4; 5M, subdivisions of BA 5; Cbl V/VI/VIIIav, cerebellar lobules V/VI/VIIIa vermis; Cd, caudate nucleus; FG, fusiform gyrus; hOC4v, ventral part of area hOC4; MOG, middle occipital gyrus; LG, lingual gyrus; MCC/PCC, middle/posterior cingulate cortex; PCu, precuneus; PF, area PF in the inferior parietal cortex; TE1.1, area TE1.1; Tp/Tpf/Tt, parietal/prefrontal/temporal subdivision of the thalamus; L, left; R, right.

Connector hubs that appeared in both patients and controls (i.e., shared connector hubs) were found in the cerebellar lobules V (left) and VI (bilateral) as well as in the left parietal and prefrontal subdivisions of thalamus, lingual gyrus (bilateral), and the right precuneus. Right Brodmann area (BA) 6 was the only shared provincial hub. Controls employed as connector hubs the left cerebellar lobule VIIIa (vermis), areas TE1.1 and hOC4v and the right parietal and prefrontal subdivisions of thalamus, which, however, did not reach hub status in the patient network. The patient network, on the other hand, employed connector hubs in left primary motor cortex, caudate nucleus, middle occipital gyrus, precuneus, right primary motor cortex, cerebellar lobule V, bilateral middle and posterior cingulate gyrus and temporal subdivision of thalamus. The control network included more provincial hubs (Figure 1B), some of which did not reach the hub status in WC network or were not even included in the network (Figure 2). However, some regions that were provincial hubs in the control network advanced to connector hubs in the patient network (left BA 4, precuneus, middle cingulate cortex). Two regions to note were nodes that were connector hubs in one network but not even included as nodes in the other network, namely the left cerebellar module VIIIa vermis (connector hub in the control network) and middle cingulate cortex (connector hub in the patient network), as shown in Figure 2.

#### Simple vs. Complex Writer's Cramp

##### Network Metrics

There was a significant difference in mean clustering coefficient in the CWC and SWC networks (CWC: 0.14 ± 0.02, SWC: 0.13 ± 0.02, *p* ≤ 0.00005), but not in mean nodal degree (CWC: 0.50 ± 0.19, SWC: 0.50 ± 0.18, *p* = 0.97), mean nodal strength (CWC: 0.09 ± 0.04, SWC: 0.09 ± 0.04, *p* = 0.22) or global efficiency (CWC: 0.16 ± 0.08, SWC: 0.16 ± 0.07, *p* = 0.75).

##### Hub Formation

**Figure 3:**
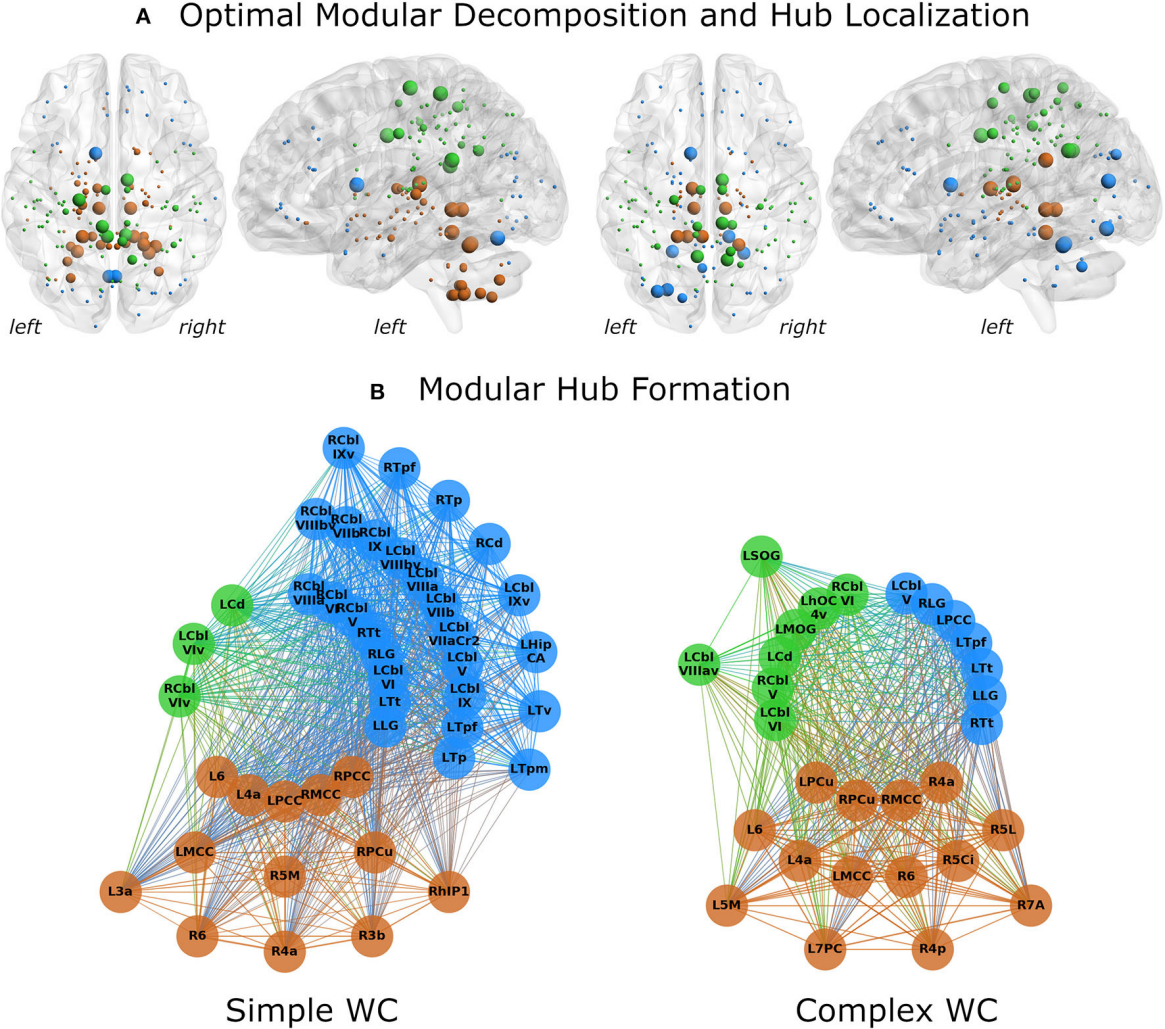
Network analysis results for simple (left) and complex (right) writer's cramp subgroups. **(A)** The optimal modular decomposition is shown by colored nodes in a 3D glass brain. Both groups had three modules: a fronto-occipital module (blue), a parietal module (green), and a subcortical module (brown). The subcortical module was larger in the SWC group, spreading into the cerebellum. The size of each node indicates its hub status, from largest to smallest: connector hubs, provincial hubs, high influence nodes, other nodes. Hub distribution in both groups was mostly restricted to the posterior part of the brain. **(B)** Hubs per module. All regions that were either hubs or high influence nodes are presented as colored nodes. Color illustrated module affiliation (as in **A**). Nodes are arranged in three circles according to their hub status: connector hubs (innermost circle), provincial hubs (intermediate circle) and high influence nodes (outer circle). Both groups recruited nearly the same number of connector hubs, but the SWC group's network contained more provincial hubs and high influence nodes. 3a/3b/6, BAs 3a/3b/6; 4a/4p, anterior/posterior part of BA 4; 5Ci/5L/5M, subdivisions of BA 5; 7A/7PC, subdivisions of BA 7; Cbl V/VI/VIv/VIIa crus II/VIIb/VIIIa/VIIIav/VIIIbv/IX/IXv, cerebellar lobules V/VI/VI vermis/VIIa crus II/VIIb/VIIIa/VIIIa vermis/VIIIb vermis/IX/IX vermis; Cd, caudate nucleus; HipCA, subdivision of the hippocampus; hlP1, intraparietal sulcus subdivision hlP1; hOC4v, ventral part of area hOC4; LG, lingual gyrus; MCC/PCC, middle/posterior cingulate cortex; MOG, middle occipital gyrus; PCu, precuneus; SOG, superior orbital gyrus; Tp/Tpf/Tpm/Tt/Tv, parietal/prefrontal/premotor/temporal/visual subdivision of the thalamus; L, left; R, right.

Splitting the patient group into two subgroups (complex vs. simple writer's cramp) showed distinct differences between their networks. In the SWC network, the subcortical module was far more spread out and included all cerebellar nodes (Figure 3A), which were part of the fronto-occipital network in all other groups. Modularization was significantly different between CWC and SWC (*pd* = 0.337, *p* < 0.0001). Moreover, both CWC and SWC networks showed significantly different modularization than the control network. Interestingly, the partition distance to the control network was larger for the SWC (*pd* = 0.347, *p* < 0.0001) than for the CWC (*pd* = 0.303, *p* < 0.0001).

Hub characteristics were also different for these networks (Figure 3B). While the SWC network had more hubs (30 vs. 25), the amount of connector hubs was nearly identical (SWC: 16; CWC: 17). The SWC network had more than three times as many high influence nodes as the CWC network (13 vs. 4).

**Figure 4:**
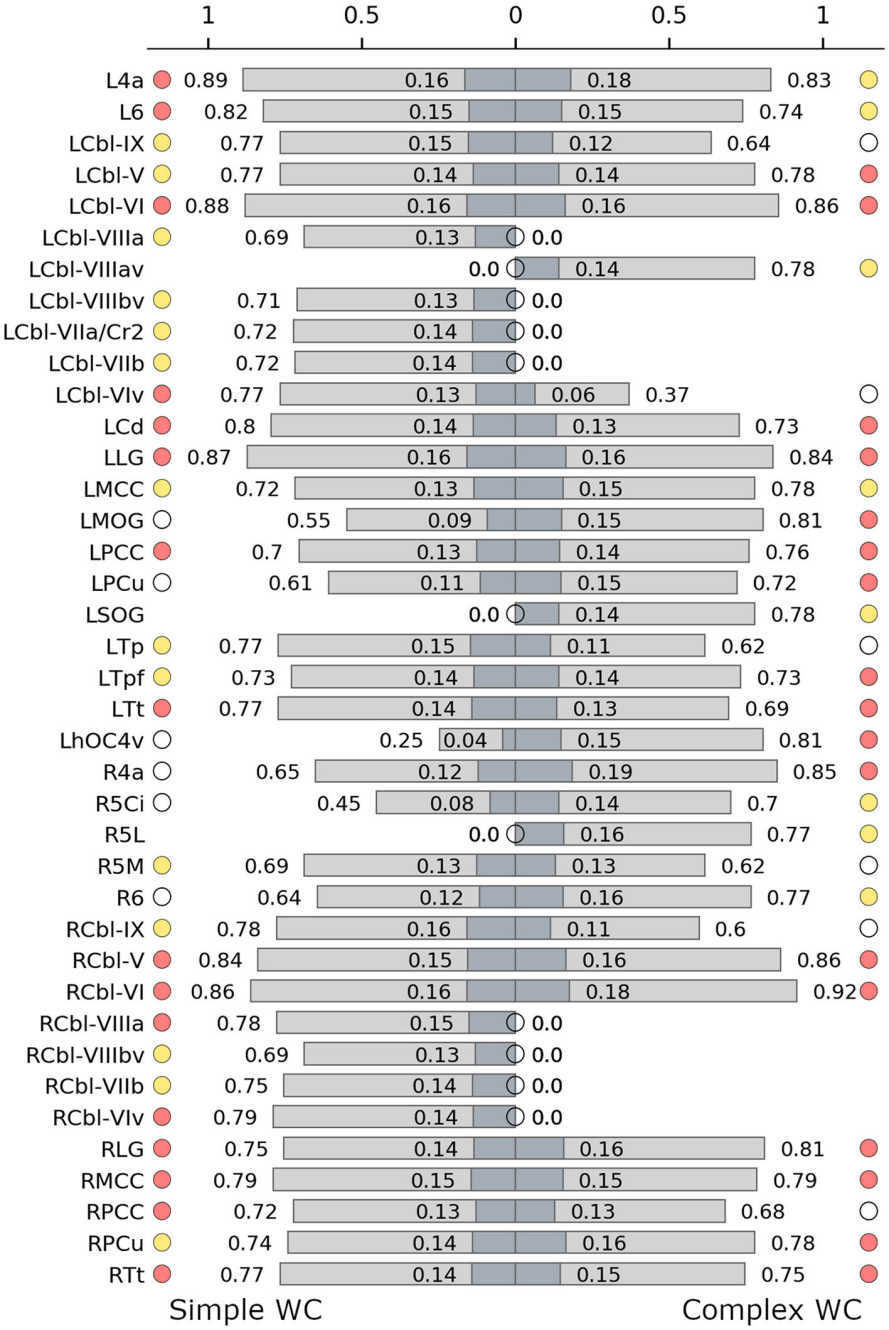
Hub characteristics of the simple **(left)** and complex **(right)** writer's cramp networks. The graph shows all nodes that were recruited as hubs in either of the two networks. For each hub, normalized nodal degree (light grey) and normalized nodal strength (dark grey) are indicated by bars. White circles denote nodes that were not hubs in the respective network, absence of circles denotes that the node was not present in the respective network. Red circles denote connector hubs, yellow circles denote provincial hubs. 6, BA 6; 4a, anterior part of BA 4; 5Ci/5L/5M, subdivisions of BA 5; Cbl V/VI/VIv/VIIaCr2/VIIb/VIIIa/VIIIav/VIIIbv/IX, cerebellar lobules V/VI/VI vermis/VIIa crus II/VIIb/VIIIa/VIIIa vermis/VIIIb vermis/IX; Cd, caudate nucleus; hOC4v, ventral part of area hOC4; LG, lingual gyrus; MCC/PCC, middle/posterior cingulate cortex; MOG, middle occipital gyrus; PCu, precuneus; SOG, superior orbital gyrus; Tp/Tpf/Tt, parietal/prefrontal/temporal/ subdivision of the thalamus; L, left; R, right.

There were 10 shared connector hubs, including bilateral lingual gyrus, the temporal subdivision of thalamus, cerebellar lobule VI, left posterior cingulate gyrus, caudate nucleus, right middle cingulate gyrus and cerebellar lobule V (Figure 4). The left middle cingulate gyrus was the only shared provincial hub.

## Discussion

In this study, we employed graph theoretical analysis to characterize functional network changes in WC patients during production of a finger-tapping task with the non-dystonic hand. Thus, we were able to investigate underlying changes in functional connectivity in WC that were not influenced by motor task performance. Our results show that, while patients exhibited an optimal modular decomposition of their neural network similar to that of healthy controls, topological differences were found in the recruitment of brain regions and in the manner these regions contribute to the network (i.e., act as relays between modules or propagate information within one module). Distinct differences could be observed in thalamic and cerebellar function and the recruitment of primary motor cortex. In a secondary analysis, we compared the functional connectivity between simple vs. complex WC and found that the network in SCW patients recruited an abnormally large number of cerebellar regions.

### Network Metrics

Our nodal elimination strategy allowed us to investigate whether the WC network recruited different brain regions compared to healthy controls. Indeed, we showed that WC patients exhibited a reduced number of regions contributing to the network. Global efficiency did not differ significantly between the two networks, indicating that the rerouting within the patient network did not impair the networks ability to process information. Furthermore, mean nodal degree and mean nodal strength did not significantly differ between groups. This might be an inherent consequence of the nodal elimination strategy, as both these metrics are influenced by the removal of the weakest connections. The network's clustering coefficient was significantly larger in patients, indicating that the patient network's nodes had a higher tendency to form functional clusters. It seems that in the patients' network information processing was achieved in a more discretely structured way.

### Modularization and Hub Characteristics

The optimal modular decomposition differed between groups and communication within and between modules was changed in the patient network. The increased number of connector hubs in the patient network indicates increased intermodular communication. This means that activity in a module influences activity in the other modules in an abnormal way. The design of the current analysis does not enable us to say whether this abnormal communication pattern is caused by the disorder or is a manifestation of compensatory mechanisms trying to make up for lost function of a module.

The analysis revealed three interesting findings that we discuss further:

First, the primary motor cortex changed its status as a provincial hub in the control network to connector hub in the patient network. Therefore, activity in the primary motor area in the patient network more strongly influenced network-wide connections. This is in line with previous investigations of the same data set, which have shown reduced inhibition of the primary motor area with simultaneous increased output to areas such as the cerebellum and the supplementary motor area (11). A similar change of hub status was reported for the primary motor cortex in hand dystonia (including WC and musicians focal hand dystonia) (19). In an EEG study, WC patients exhibited disruption of intracortical inhibition during a writing-from memory task (39). While EEG does not have the spatial resolution to pinpoint this finding to the primary motor cortex, it supports the notion that inhibitory connections are impaired in WC.

Second, cerebellar recruitment was reduced in the patient network to only two-third of the control network's recruitment. This indicates cerebellar dysfunction, which is in line with previous research on cerebellar involvement in dystonia (40–47). Previous resting-state research on task specific focal dystonias, including WC, has shown abnormal cerebellar integration (18): While healthy participants were found to have a single, highly integrated module comprising the basal ganglia and the cerebellum, patients had multiple separate modules instead. The authors believe this sign of altered functional interaction between the basal ganglia and the cerebellum to be the base for further network abnormalities and the development of dystonic symptoms. Furthermore, Fuertinger and Simonyan (17) found task-related altered cerebellar function in patients with laryngeal dystonia. Similar to the study on task specific focal dystonias, they report a breakdown of the healthy basal ganglia-cerebellar module into separate modules in patients. Taken together, this indicates that the cerebellum might play a crucial part in the common pathophysiology of task-specific dystonia. Furthermore, in a lesion network mapping study, it was shown that while not all lesions causing cervical dystonia are located in the cerebellum, they are all located within a cerebellar network (48).

Last, the thalamus featured a large number of connector hubs in both control and patient networks, but while in the control network this was a bilateral pattern, in the patient network the left hemispheric thalamus was more prominent. Fuertinger and Simonyan (17) have shown abnormal functional integration of the thalamus in patients with laryngeal dystonia. They report the left thalamus to act as a transmitter to the basal ganglia in dystonic patients. The left thalamus was also found to play a greater communicative role in task-specific focal dystonia patients: Their network included the left thalamus as a connector hub, which was not the case for the healthy control network. It has been shown that the thalamus acts as a relay station between other brain regions and plays a role in integrating information across cortical networks (49–51). As the thalamus passes information from the cerebellum and the basal ganglia to the motor cortex (52), abnormal thalamic recruitment might be a disorder-promoting network change.

### Simple vs. Complex Writer's Cramp: Preliminary Results

In an exploratory analysis due to low number of patients in each group, we investigated differences between the networks of patients with simple vs. complex WC. Only the mean clustering coefficient differed between these groups, showing that patients with CWC had a more segregated network. Modular decomposition indicated that nodal interaction differs in SWC and CWC from the normal state, and that the difference is more pronounced in the SWC group. In the complex form of the disease, when patients have developed problems in other fine motor tasks, the optimal modular decomposition is more similar to that of controls. One possible explanation is that increased activity and connectivity in early disease stages could be a manifestation of compensatory mechanisms. We argue that since cerebellar activity is increased in simple writer's cramp, when patients have less diverse symptoms, and decreases in complex writer's cramp, when symptoms spread, that this early increase of activity could be compensatory. In the advanced stage of the disease, this compensatory activity decreases and more wide-spread symptoms occur. The finding that the cerebellar recruitment is increased in SWC (as shown by the increased numbers of provincial and connector hubs in the cerebellum) but not in CWC supports this assumption. However, in our study we did not investigate longitudinal data. Therefore, our findings are only indicative and do not provide proof of this interpretation. We suggest further research in this direction, including a larger patient sample to be studied longitudinally. Thereby, the effects of disease progression (leading from simple to complex WC) could be more directly assessed.

## Conclusion

Our graph theoretical analysis of the functional brain networks of WC patients demonstrated several changes in network functionality as compared to healthy controls. By analyzing whole-brain networks we were able to show that information processing is achieved in a more discretely structured way in WC patients. Furthermore, our hub analysis revealed abnormal connectivity of three key regions, which have been previously reported as sites of dystonia related alterations: The primary motor cortex influence on other regions was increased in WC patients, suggesting a lack of inhibition. The cerebellum was less connected than in healthy controls, indicating cerebellar dysfunction. Lastly, thalamic recruitment was more left-lateralized in patients, which could lead to alterations in how information is relayed from the cerebellum and the basal ganglia to the motor cortex. As we employed a paradigm that did not invoke dystonic symptoms in patients, we have shown that the patients' functional connectivity is changed even in the absence of symptoms. Therefore, dystonia seems to cause persistent changes in the whole-brain functional network.

## Data Availability Statement

The raw data supporting the conclusions of this article will be made available by the authors, without undue reservation.

## Ethics Statement

The studies involving human participants were reviewed and approved by the Ethics Committee of Christian Albrechts University, Kiel, Germany (A176/09). The patients/participants provided their written informed consent to participate in this study.

## Author Contributions

JS, KS, KW, and KZ contributed to the design of the research. AK, IT, and KZ obtained the data. JS, KS, and KW analyzed the data. KS contributed unpublished analytic tools for data analysis. JS wrote the first draft of the manuscript. All authors edited the manuscript and approved its final version.

## Funding

KZ has received research support from an intramural grant from the Christian-Albrechts University of Kiel, from the Benign Essential Blepharospasm Foundation, and with an unrestricted grant from Ipsen. KS receives funding from the National Institutes of Health (R01NS088160, R01DC011805, and R01DC012545), the Department of Defense (W911NF1810434), Amazon Web Services, and Mass General Brigham Innovation. The funders were not involved in the study design, collection, analysis, interpretation of data, the writing of this article or the decision to submit it for publication.

## Conflict of Interest

KW serves as a consultant for BIAL. KZ reports speaker's honoraria from Bayer Vital GmbH, AbbVie Allergan and Merz outside the submitted work. She has served as a consultant and received fees from Merz, Ipsen and the German Federal Institute for Drugs and Medical Devices (BfArM). KS serves on the Scientific Advisory Board of the Tourette Association of America. The remaining authors declare that the research was conducted in the absence of any commercial or financial relationships that could be construed as a potential conflict of interest.

## Publisher's Note

All claims expressed in this article are solely those of the authors and do not necessarily represent those of their affiliated organizations, or those of the publisher, the editors and the reviewers. Any product that may be evaluated in this article, or claim that may be made by its manufacturer, is not guaranteed or endorsed by the publisher.
