# Supplementary figures and images for: Functional Neural Networks in Writer's Cramp as Determined by Graph-Theoretical Analysis
Source: Front Neurol. 2021 Nov 23;12:744503. doi: 10.3389/fneur.2021.744503 (PMC8650489; doi:10.3389/fneur.2021.744503)

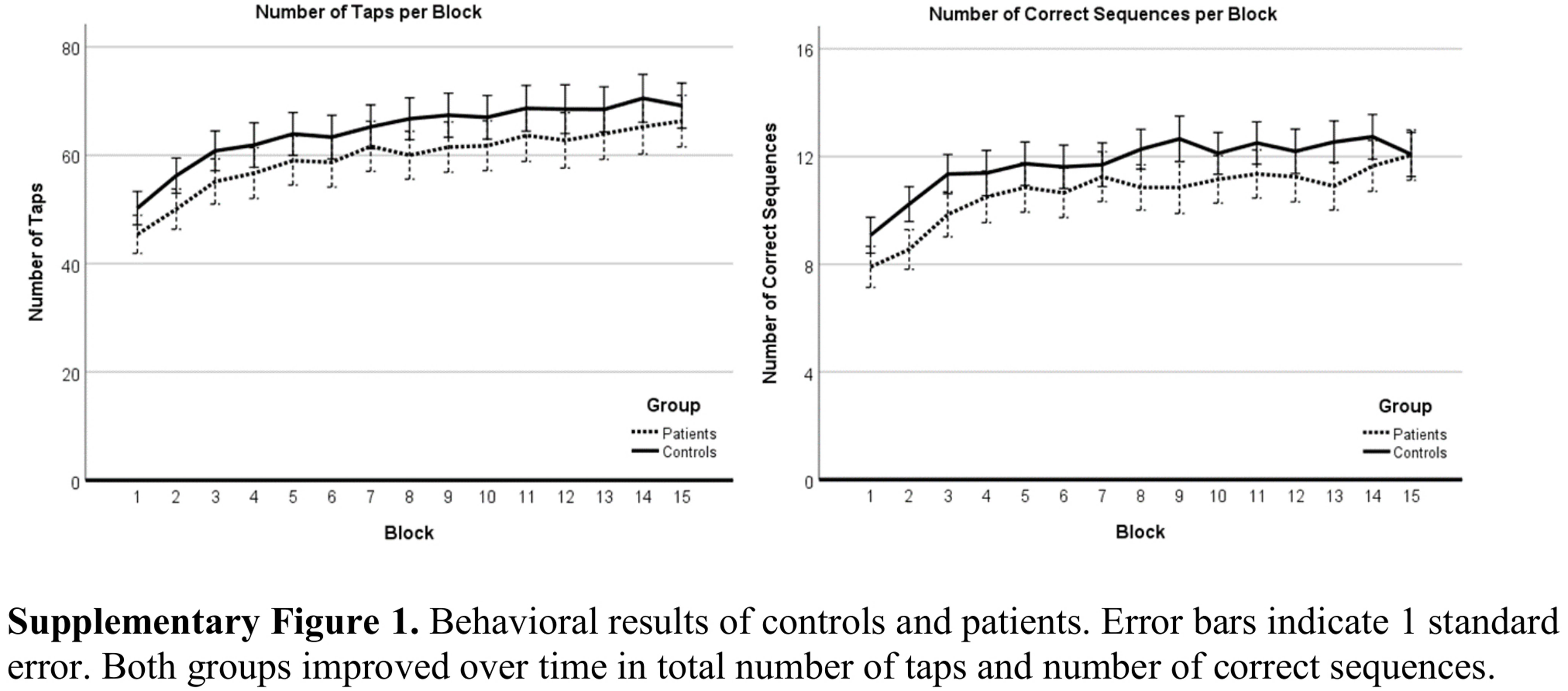

Supplement: Supplementary file 1 [file Image_1.JPEG]
